# The Public Services

**Published:** 1912-09-07

**Authors:** 


### THE PUBLIC SERVICES.

### THE NAVY MEDICAL DEPARTMENT.

This Service is only open to the registered graduate of European birth between 21 and 28 years of age. He must declare—(1) His age and date and place of birth; (2) that he is of pure European descent, and the son either of natural-born British subjects or of parents naturalised in the United Kingdom; (3) that he labours under no mental or constitutional disease or weakness nor any other imperfection or disability which may interfere with the most efficient discharge of the duties of a medical officer in any climate; (4) that he is ready to engage for general service at home or abroad, as required; (5) whether he holds, or has held, any commission or appointment in the Public Services; (6) that he is registered under the Medical Act, giving the date of his resignation as a medical student, or of his beginning professional study; (7) whether he has previously been examined for entry in the Naval Service, and, if so, when. He must pass an Entrance examination, and gain at least one-half of the possible marks, and he must satisfy the physical requirements of the Medical Board.

The examination will consist of the following subjects: (*a*) Medicine, including medical pathology and therapeutics; (*b*) surgery, including surgical pathology and clinical surgery. The examination is partly written and partly practical, marks being allotted as follows: 400 each, paper, clinical, and oral, for Medicine and Surgery. No candidate shall be considered eligible who obtains less than 50 per cent. of marks in each subject. The examination is held in London, and occupies four days. The course of instruction for acting surgeons will be six months in duration, and the first two months will be passed at the Naval Medical School at Greenwich. This period will be devoted to the study of tropical medicine, bacteriology, and clinical pathology, hygiene and skiagraphy. The remaining four months will be passed at Haslar, and will be devoted to the study of naval hygiene, recruiting, physical training, diving, and submarine work, etc. A medical officer (fleet surgeon) will be retained at Haslar to superintend these studies and fill the post of lecturer on naval hygiene.

The post-graduate instruction of naval medical officers will consist of a six months' course prior to advancement to staff surgeon, which is compulsory for all surgeons, and will be followed by an examination before the Medical Examining Board in London, the successful passing of which will be a qualification for promotion to the rank of staff surgeon. The course is to be taken when a surgeon has between four and a-half and six and a-half years' seniority, and as near the date when the latter period is completed as the exigencies of the Service admit. The courses will take place twice a year.

The subjects of study and examination will be:—Compulsory: (*a*) Clinical medicine and clinical surgery; (*b*) operative surgery; (*c*) anæsthetics (practical); (*d*) ophthalmology; (*e*) clinical pathology; (*f*) hygiene. Optional: (*g*) Bacteriology; (*h*) throat, nose, and ear diseases; (*i*) skiagraphy. A surgeon who fails to obtain a pass will be allowed a second trial, but should he again be unsuccessful he will be compulsorily retired on attaining eight years' seniority, with such gratuity as the Admiralty may see fit to grant, but not exceeding £500. Instruction in bacteriology and clinical pathology, hygiene, and skiagraphy will be given at the Naval Medical School; the remaining subjects will be studied at the "Dreadnought" Hospital and other civil hospitals in London, as may be arranged from time to time by the Medical Director-General of the Navy.

The surgeons going through the course will be accommodated at the Royal Naval College, Greenwich, and will be under the general control of the president. Their instruction throughout will be under the immediate supervision of the professor of hygiene appointed in charge of studies.

### THE ROYAL ARMY MEDICAL CORPS.

A candidate for a commission in the Royal Army Medical Corps must be 21 years and not over 28 years of age at the date of the commencement of the Entrance examination. He must be registered under the Medical Acts in force in the United Kingdom. Before being permitted to proceed to the Entrance examination, the candidate is required to attend at the War Office about two days before the commencement for the purpose of being interviewed and undergoing physical examination. The Entrance examination is held twice a year in London—January and July—a fee of £1 being charged for admission thereto. It lasts about three days. The examination is of a clinical, written, practical, and oral character. It consists of: A commentary on a case in medicine; a commentary on a case in surgery; examination and report on a clinical medical case; examination and report on a clinical surgical case; orals in clinical medicine and clinical surgery and pathology; orals in medical pathology and surgical operations and bandaging (including surgical instruments and appliances). A candidate who is successful at the Entrance examination is appointed a lieutenant, and is then required to attend at the Royal Army Medical College, London, and afterwards at the Royal Army Medical Corps School of Instruction, Aldershot, for the purpose of qualifying in the following subjects before his commission can be confirmed:—Royal Army Medical College: Military surgery, tropical medicine, hygiene, pathology, and organisation of military hospitals, and principles of governing medical charge of troops. Royal Army Medical Corps School of Instruction: Regimental duties, drill and field training.

### THE INDIAN MEDICAL SERVICE.

Candidates must be natural-born subjects of His Majesty, of European or East Indian descent, between 21 and 28 years of age at the date of the examination, of sound bodily health, and in the opinion of the Secretary of State for India in Council in all respects suitable to hold commissions in the Indian Medical Service. They may be married or unmarried. They must possess, under the Medical Acts in force at the time of their appointment, a registrable qualification to practise both medicine and surgery in Great Britain and Ireland.

The physical fitness of each candidate will be determined by a Board of Medical Officers, who are required to certify that his vision is sufficiently good to enable him to pass the tests laid down by the Regulations. The candidate will be examined by the Examining Board in the following subjects:

(1) medicine, including therapeutics; (2) surgery, including diseases of the eye; (3) applied anatomy and physiology; (4) pathology and bacteriology; (5) midwifery and diseases of women and children; (6) materia medica, pharmacology, and toxicology. The examination in medicine and surgery will be in part practical, and will include operations on the dead body, the application of surgical apparatus, and the examination of medical and surgical patients at the bedside. No syllabus is issued in the subjects of the examination which will be conducted so as to test the general knowledge of the candidate in these subjects. No candidate shall be considered eligible who shall not have obtained at least one-third of the marks obtainable in each of the above subjects and one-half of the aggregate marks for all the subjects.

After passing examination, the successful candidates will be required to attend a course of practical instruction at the Army Medical School and elsewhere, as may be decided, in: (1) hygiene; (2) military and tropical medicine; (3) military surgery; (4) pathology of diseases and injuries incidental to military and tropical service. This course will be of not less than four months' duration, and at its conclusion candidates will be required to pass an examination in the subjects taught therein.

### THE COLONIAL MEDICAL SERVICE.

Full details regarding this service and the advantages it offers to newly qualified men may be obtained on application to the Private Secretary, Colonial Office.

